# Variation of PD-L1 expression in locally advanced cervical cancer following neoadjuvant chemotherapy

**DOI:** 10.1186/s13000-020-00977-1

**Published:** 2020-06-03

**Authors:** Yun Liang, Minghua Yu, Caiyun Zhou, Xiaojun Zhu

**Affiliations:** 1 Department of Surgical Pathology, the Affiliated Women’s Hospital, School of Medicine, Zhejiang University, Hangzhou, 310006 Zhejiang Province China; 2 Department of Gynaecology and Obstetrics, the Affiliated Women’s Hospital, School of Medicine, Zhejiang University, Hangzhou, 310006 Zhejiang Province China

**Keywords:** Squamous cell carcinoma, Cervix, Tumor infiltrating lymphocytes, PD-L1, Neoadjuvant chemotherapy

## Abstract

**Background:**

High Programmed death ligand 1 (PD-L1) expression are thought to be necessary to PD-1/PD-L1 axis blockades in many tumors. The aim of the study was to explore the variation of PD-L1 expression after neoadjuvant chemotherapy (NAC) in cervical squamous cell carcinoma (SCC) and its clinical implications.

**Methods:**

A total of 142 paired SCC specimens before and after platinum-based NAC were obtained from cervical cancer patients. The expression of PD-L1 and CD3+, CD4+, CD8+ tumor infiltrating lymphocytes (TILs) was detected by immunohistochemistry and the association between TILs, chemotherapy response, clinical outcome and PD-L1 expression was evaluated.

**Results:**

The fraction of patients with high PD-L1 expression was significantly increased from 32.4 to 46.5% after NAC (χ2 = 5.897, *p* = 0.015), while the increase of CD3+, CD4+, CD8+ TILs was not significant. High PD-L1 expression was not associated with CD3+, CD4+, CD8+ TILs before NAC, however CD8+ TILs infiltration was positively associated with high PD-L1 expression after NAC (r = 0.205, *p* = 0.014). The decreased PD-L1 expression was more observed in patients with clinical response to NAC (χ2 = 6.890, *p* = 0.009). A longer DFS was seen in patients with decreased PD-L1 expression than those with elevated or stable PD-L1 expression (*p* = 0.048, 95% CI: 0.091–0.987), while the difference was not significant in multivariate analysis (*p* = 0.113, 95% CI: 0.108–1.266).

**Conclusions:**

Cisplatin based chemotherapy can increase PD-L1 expression in cervical cancer. The increased PD-L1 expression and a lymphocyte predominant microenvironment after chemotherapy provide a rational for use of PD-1/PD-L1 axis-inhibitor in the neoadjuvant setting.

## Background

Cervical cancer is the fourth commonest malignancies in women worldwide and nearly 40% of tumors are diagnosed at FIGO stage IB2-IIA2 [1]. As one of the adjuvant treatments of cervical cancer, NAC reduces tumor volume, increases tumor resectability, and eliminates micro-metastases. NAC followed by radical surgery offers a promising treatment modality for patients with locally advanced cervical cancer [2]. For only patients who responded well to NAC have survival benefit, it is necessary to combine new treatment strategies to enhance therapeutic efficacy. Recently, Immune checkpoint inhibitors, targeting PD-L1/PD-1 have yielded great achievements in some solid cancers. Several ongoing studies are testing if addition of an immune checkpoint inhibitor to chemotherapy could improve survival [3–5].

In the past, chemotherapy was thought to induce cell death through cytotoxic effect without a major impact on the immune system. However, recent data suggested that tumor cell injury from chemotherapy can change the tumor microenvironment [6]. PD-L1 is an immune inhibitory molecule expressed in tumor cells and some immune cells. PD-L1 suppresses the activation of T cell upon binding to its receptor, PD-1. High PD-L1 expression is believed the prerequisite for PD-L1/PD-1 immunotherapy. However, not all the patients with high PD-L1 expression have response to immune checkpoint treatment and the degree of tumor infiltrating lymphocytes (TILs) in the tumor microenvironment are also correlated with the clinical outcomes of anti-PD-1/PD-L1 therapies [7].

We hypothesized that cisplatin based chemotherapy could alter the expression of TILs and PD-L1, which would significantly influence the efficiency of immunotherapy. In the present study, we investigated the dynamics of PD-L1 expression and CD4, CD3, CD8+ TILs to ascertain whether cervical cancer patients could benefit from immunotherapy following primary treatment.

## Methods

### Patients selection and clinical information

This study was a retrospective analysis of patients consecutively collected at the Women’s Hospital, School of Medicine, Zhejiang University, China, between January 2011 and December 2016. All patients had 2014 FIGO stage IB2-IIA2 (≥ 4 cm in diameter) cervical squamous cell carcinoma (SCC) and underwent platinum-based NAC (paclitaxel 175 mg/m2 [3-h infusion] + cisplatin 75 mg/m2, every 3 weeks, for 2 cycles) prior to surgery. Pre-NAC specimens were obtained by biopsy at the time of initial diagnosis, and post-NAC specimens were obtained at the time of surgery. Patients who failed to complete the planned cycles of NAC were excluded from the study. Clinical response to chemotherapy was determined according to WHO clinical tumor response criteria [8]. Complete response (CR) was defined as the disappearance of all known tumors, partial response (PR) as 50% or more decrease in the total tumor volume, no change (NC) as a less than 50% decrease in total tumor or has a less than 25% increase in the size of measurable lesions, and progressive disease (PD) as a 25% or more increase in the size of measurable lesions. The patients with CR or PR is regarded as clinically effective, while NC or PD as non-effective.

### Immunohistochemical analysis of PD-L1 expression and TILs

Sections including biopsy specimens before NAC and surgical specimens after NAC were reviewed to confirm the histopathological diagnosis and adequacy of specimens for immunohistochemistry analysis. Immunohistochemical staining were performed on 5-μm-thick sections of formalin-fixed, paraffin-embedded tissue, using 2-step En vision method according to the manufacturer’s instructions and visualized with 3-diaminobenzidine tetrachloride (Sigma, St Louis, MO). The sections were incubated with antibodies against PD-L1(clone ZR3; Zeta, USA; 1:200 in dilution), CD8(clone SP16; Thermo, USA; 1:200 in dilution), CD3(clone LN10; Thermo, USA; 1:100 in dilution),CD4(clone 4B12; Dako, Denmark; 1:100 in dilution). Appropriate positive and negative controls were stained concurrently.

PD-L1 expression score on tumor cells or stromal immune cells was counted by scoring the proportion of membranous positive cells over the total number of cells, and grouped the scores into three categories based on cutoff in previous studies (0,< 1%; 1, 1–50%; and 2,≥50%) [9, 10]. When ≥1% membranous staining, it was defined as positive and ≥ 50% membranous staining defined as high expression. The number of CD3+, CD4+ and CD8+ TILs were counted in 10 high-power fields (five tumor areas and five surrounding peritumoral stroma) of highest density. CD3+, CD4+ and CD8+ TILs densities were defined as positive staining numbers per square millimeter. The immunohistochemical staining was independently evaluated by two of the authors who were blinded to the clinical data.

### Statistical analysis

Statistical analyses were performed by SPSS 21.0 software (Chicago, IL, USA). The Chi-squared test was used for evaluating the relationship between clinicopathologic characteristics, chemotherapy response and PD-L1 expression. Intra-class correlations (ICCs) were calculated to investigated inter-reader concordance of PD-L1 expression score. The Wilcoxon signed-rank test was used to compare CD4+, CD8+, CD3+ TILs, or PD-L1 expression score between matched pre- and post-NACT specimens. Spearman’s correlation was used to examine the relation between tumor PD-L1 score and CD4+, CD8+, CD3+ TILs density, as well as the PD-L1 score between tumor and stromal immune cells. The patient survival was assessed by the Kaplan-Meier method. Univarate and multivariate regression was performed using the Cox proportional hazards model. All tests were two-tailed with a *P* value < 0.05 considered statistically significant. Disease free survival (DFS) was calculated from the date of initial diagnosis to either the date of disease or the date of last contact.

## Results

### Patient characteristics

A total of 142 patients were included for the final analysis. The median age of the patients at diagnosis was 45, ranging for 24 to 67 years old. Ninety-six patients were in FIGO IB2 stages, and forty-six patients were in FIGO IIA2 stages. Histologically, all cases were squamous cell carcinoma, with 115 well or moderately differentiated. Lymph node metastasis of tumor was detected in 14 cases (9.9%). HPV DNA testing results were available in 117 cases including 111 positive and 6 negative results. After 2 circles of NAC, 108(76.1%) patients achieved clinical response. With a median follow-up of 68 months (range 27–92 months), 30 patients (21.1%) had recurrent disease after a median time of 22 months (range 9–52 months), 13 patients (9.2%) had died with a median survival time of 38 months (range 18–53 months).

### PD-L1 expression and the correlation with clinicopathological features before NAC

**Fig. 1:**
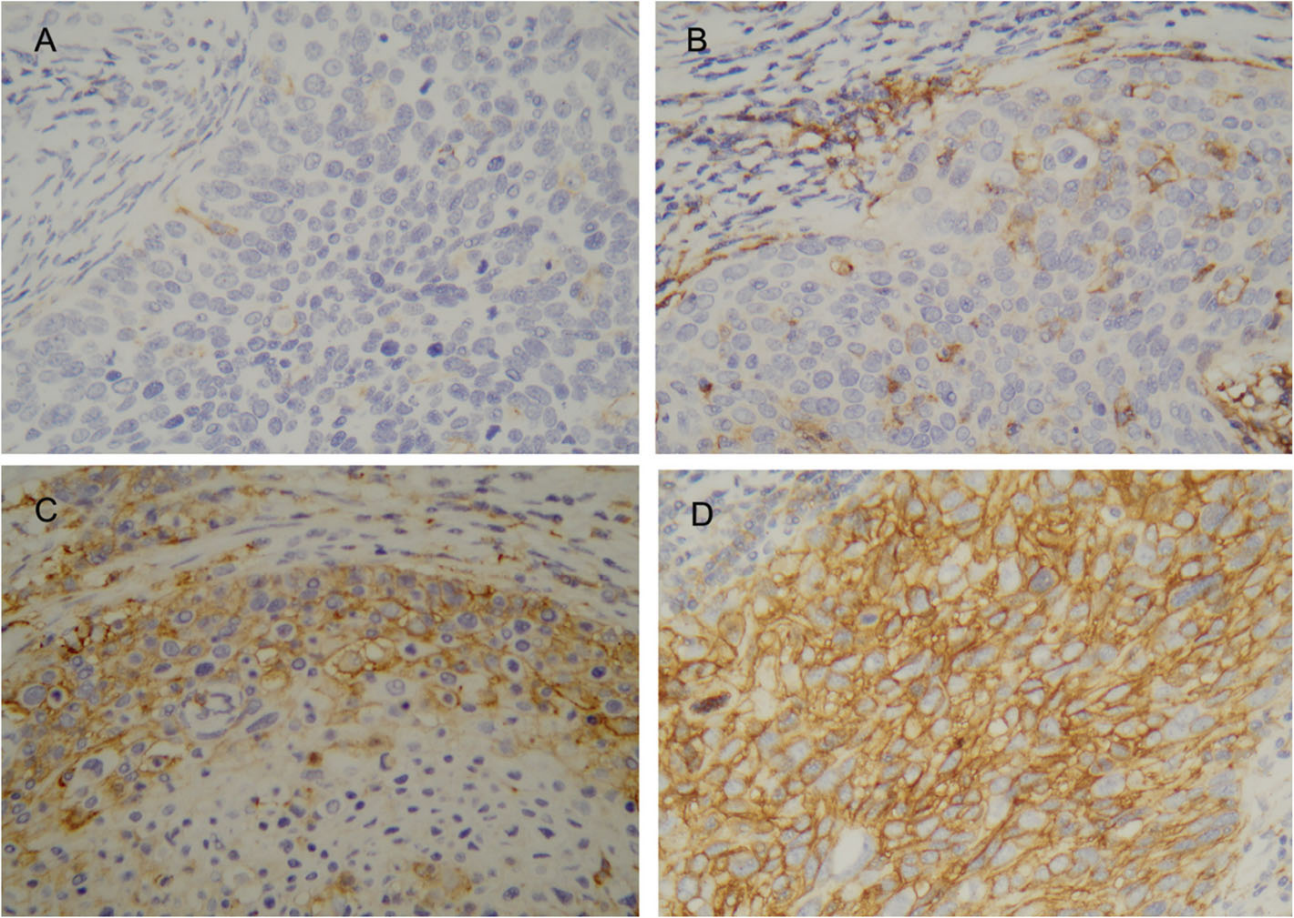
Immunohistochemical staining patterns for PD-L1 on tumor cell. Representative negative expression (A), patchy expression (B), marginal(C), and diffuse expression(D) for PD-L1

PD-L1 expression was present in tumor cells with a patchy, marginal or diffuse staining pattern (Fig. 1). Using a 1% threshold, tumor PD-L1 expression was observed in 124 (87.3%) cases*,* and 46 (32.4%) had high PD-L1 staining (> 50%). PD-L1 expression was also detected in stromal immune cells with 137 (96.5%) patients positive using a 1% threshold and 21 (14.8%) high PD-L1 staining (> 50%). A moderate PD-L1 expression correlation was seen between tumor cells and stromal immune cells(r = 0.569, *p* = 0.001). Inter-reader agreement was high for PD-L1 tumor cell staining results (ICC = 0.926, 95%CI: 0.898–0.946). In contrast, there was more variation in the percentage of PD-L1 stained immune cells between individual readers (ICC = 0.517, 95%CI: 0.385–0.628). So we only analyze PD-L1 expression in tumor cells in the following research. The correlations between high tumor PD-L1 expression and clinicopathological features are shown in Table 1. There were no associations between high tumor PD-L1 expression and clinical features, except that the high tumor PD-L1 expression was more commonly seen in younger patients (χ2 = 4.631, *p* = 0.031)*.*

**Table 1:** Correlations between clinicopathological characteristics and PD-L1 expression before NAC, after NAC, as well as the change of PD-L1

	Age		FIGO stage		Histological grade		Clinical response		Lymph node metastasis		HPV status	
	< 40	≥40	Ib2	IIa2	I-II	III	CR	Non-CR	Yes	No	+	_
PD-L1 before NAC												
Low	16	80	68	28	80	16	71	25	9	87	36	3
High	15	31	28	18	35	11	37	9	5	41	75	3
p value (χ2)	0.031 (4.631)		0.235 (1.410)		0.303 (1.060)		0.397 (0.716)		0.780 (0.078)		0.374 (0.791)	
PD-L1 after NAC												
Low	18	58	52	24	62	14	61	15	5	71	63	4
High	13	53	44	22	53	13	47	19	9	57	48	2
p value (χ2)	0.566 (0.329)		0.824 (0.050)		0.847 (0.037)		0.207 (1.589)		0.159 (1.980)		0.633 (0.228)	
PD-L1 change												
increase/stable	20	85	73	32	87	18	74	31	12	93	80	3
decrease	11	26	23	14	28	9	34	3	2	35	31	3
p value (χ2)	0.176 (1.829)		0.411 (0.677)		0.338 (0.916)		0.009 (6.890)		0.291 (1.117)		0.246 (1.345)	

### Change in tumor PD-L1 expression following NAC

**Fig. 2:**
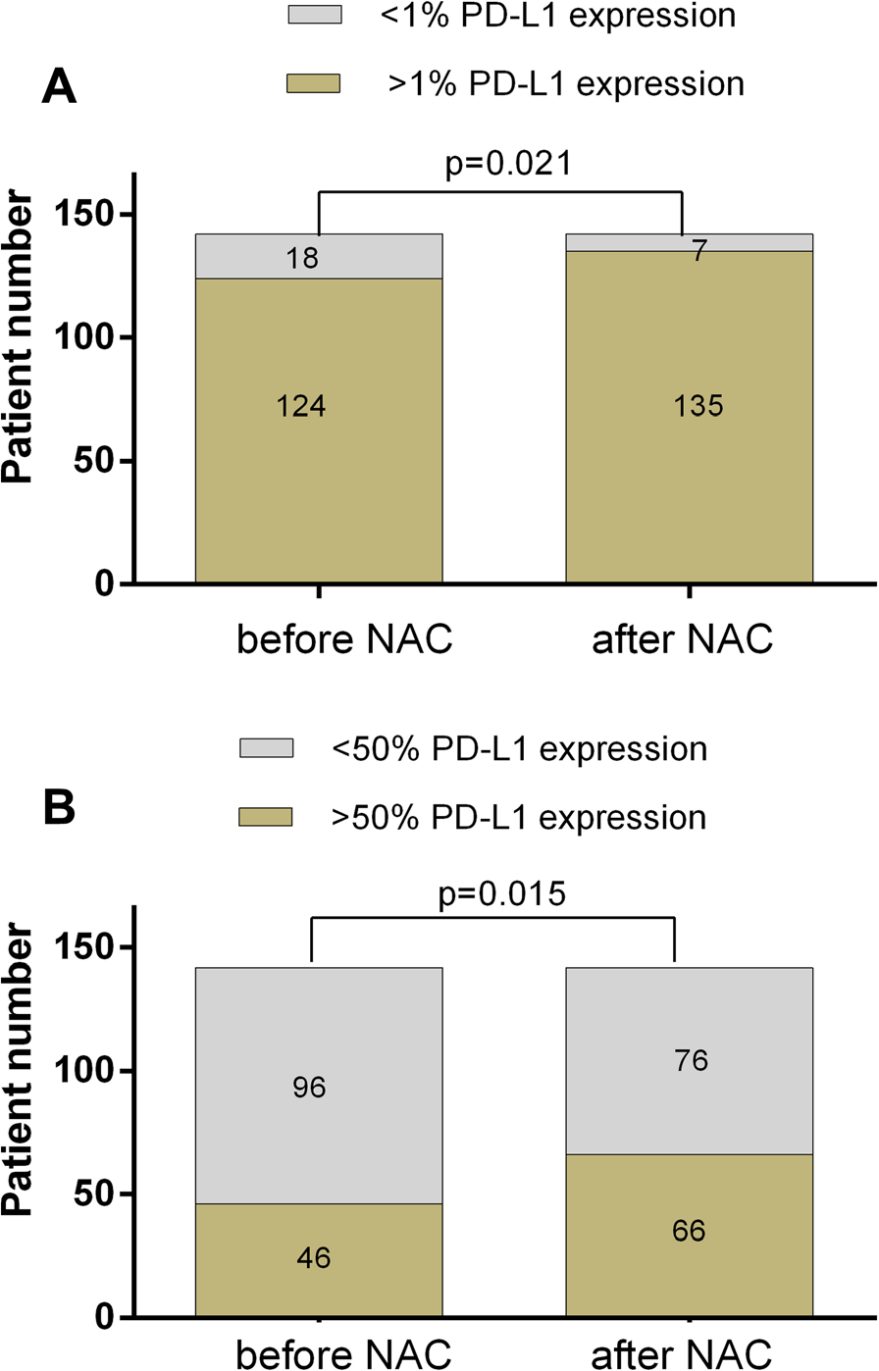
Distribution of PD-L1 expression before and after NAC. More patients had PD-L1 expression with a threshold > 1% (A) and > 50% (B) after NAC

PD-L1 expression changed significantly after NAC. One hundred and thirty-five (95.1%) patients had PD-L1 expression with a threshold > 1% (χ2 = 5.307, *p* = 0.021) (Fig. 2.A), and 66 (46.5%) patients had high PD-L1 expression (χ2 = 5.897, *p* = 0.015) (Fig. 2.B) after NAC. For paired pre- and post- samples, 67 patients showed elevation, 36patients showed reduction and 39 patients showed no variation. Further assessment showed that high tumor PD-L1 expression after NAC had no relation with clinical characteristics while the decline of PD-L1 after NAC was more frequently observed in patients who had clinical response to chemotherapy (χ2 = 6.890, *p* = 0.009),(Table.1).

### CD4+, CD8+ and CD3+ TILs density before and after NAC and its association with PD-L1 expression

**Fig. 3:**
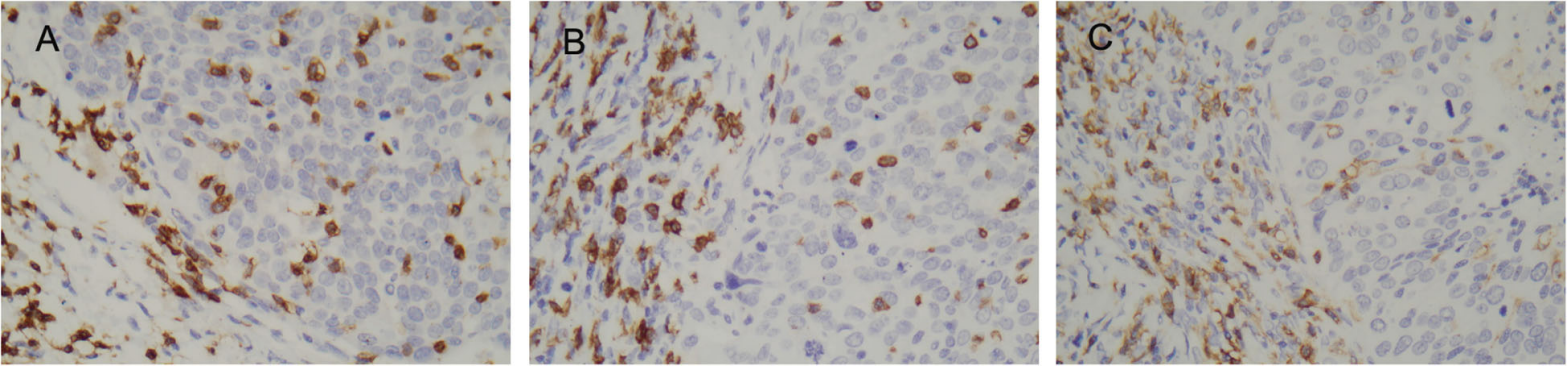
Immunohistochemical staining for TILs. CD3(A), CD8(B), and CD4(C) were present in both tumor area and surrounding peritumoral stroma

TILs were detectable in all tumor samples and present both in cancer epithelium and surrounding stroma (Fig. 3). Before NAC, the median CD4+, CD8+ and CD3+ TILs density was 175.27, 217.22, 232.77 respectively. The correlation between the density of CD4+, CD8+, CD3+ TILs and high PD-L1 expression was weak and nonsignificant (r = 0.085, *p* = 0.314; r = 0.020, *p* = 0.731; r = 0.084, *p* = 0.320). After NAC, there was a slight increase of CD4+, CD8+ and CD3+ TILs, with median 204.61, 234.10, 243.03 respectively, but this difference was not significant (*p* = 0.292; *p* = 0.132; *p* = 0.798) (Supplement Fig. 1). No significant correlation between CD3+, CD4+ TILs and high tumor PD-L1 expression was seen after NAC (r = 0.045, *p* = 0.595; r = 0.057, *p* = 0.504). However, the CD8+ TILs infiltration was associated with tumor PD-L1 expression at this time(r = 0.205, *p* = 0.014).

### Survival analysis

**Fig. 4:**
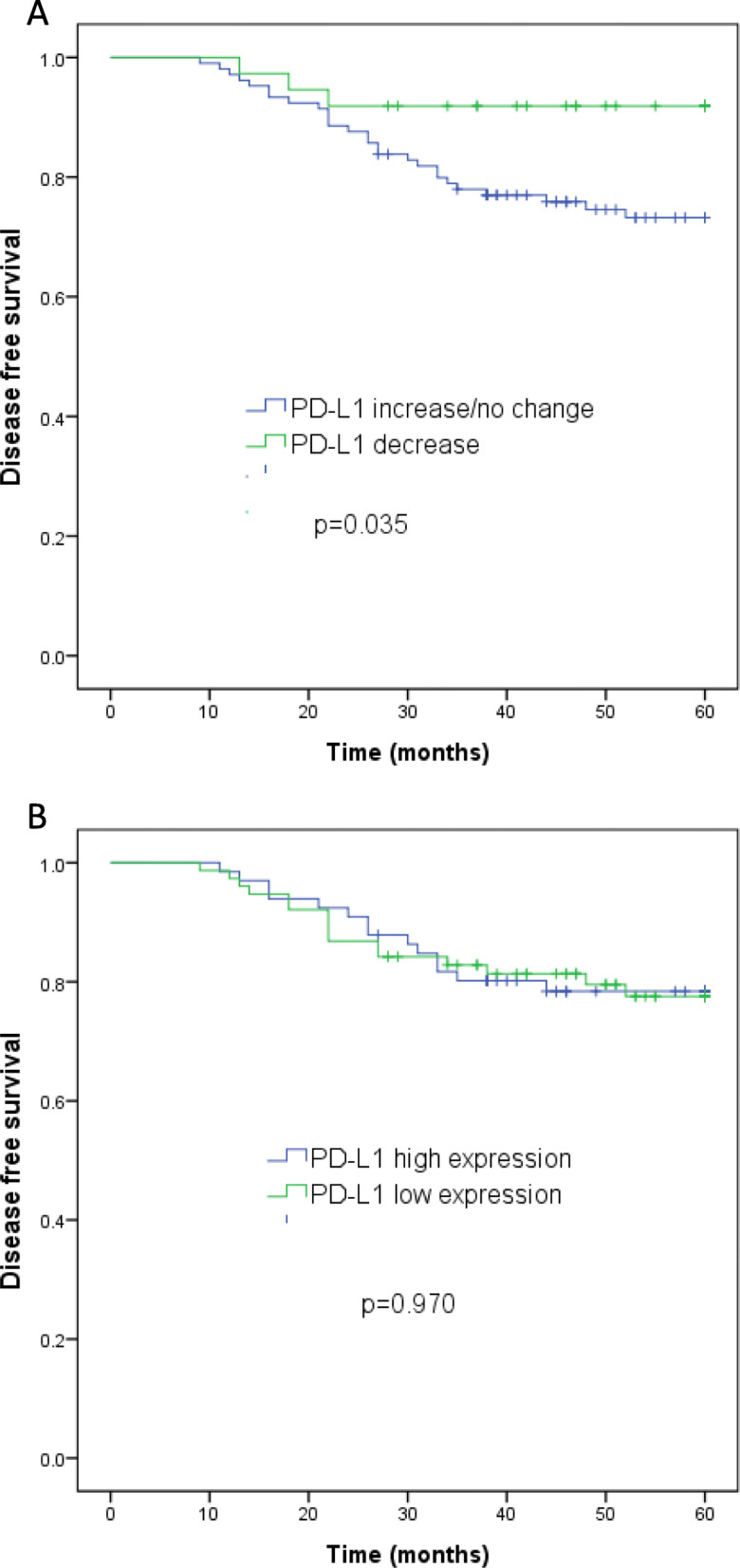
Survival analysis of cervical cancer patients. Kaplan-Meier curves for disease free survival of cervical cancer patients classified according to PD-L1 change (A) and PD-L1 expression after NAC (B)

The association of PD-L1 expression and TILs density with survival was assessed. Kaplan-Meier analysis demonstrated that high or low PD-L1 expression level in residual tumor cells did not show any significant impact on DFS (mean DFS 52.6 vs. 52.4 months, *p* = 0.970). However, a longer DFS was seen in patients with decreased PD-L1 expression than those with elevated or stable PD-L1 expression (mean DFS 56.6 vs. 51.1 months, *p* = 0.035) (Fig. 4.A,B). As for TILs, patients with higher CD8+ TILs in residual tumors had a better DFS than those with lower CD8+ TILs (mean DFS 54.9 vs. 50.1 months, *p* = 0.046). DFS did not differ significantly between patients with different density of CD3+ (mean DFS 53.7 vs. 51.3 months, *p* = 0.420) or CD4+ (mean DFS 52.7 vs. 52.3 months, *p* = 0.668) TILs after NAC, (Supplement Fig. 2).

Univariate and multivariate analysis of DFS were summarized in Table 2. In univariate analysis, FIGO stage, Lymph node metastasis, clinical response to chemotherapy, CD8+ TILs density after NAC and the decreased PD-L1 were prognostic factors for DFS. In multivariate analysis, FIGO stage remained the only independent prognostic factor for DFS (*p* = 0.024).

**Table 2:** Univaraite and multivariate analysis of DFS in cervical cancer patients

	DFS (univariate analysis)			DFS (multivariate analysis)		
	HR	95%CI	p	HR	95%CI	p
Age (< 40/≥40)	1.158	0.473–2.834	0.748			
Histo-grade (I-II/III)	1.146	0.468–2.804	0.765			
Figo stage (Ib2/ IIa2)	2.456	1.199–5.033	**0.014**	2.386	1.119–5.089	**0.024**
Clinical response (CR/non-CR)	0.441	0.214–0.909	**0.026**	0.531	0.239–1.180	1.120
Lymph-node metastasis (yes/no)	2.702	1.104–6.616	**0.030**	1.251	0.448–3.497	0.669
PDL1 after NAC (High/Low)	0.893	0.624–1.279	0.537			
PDL1 (increase or stable/decrease)	0.299	0.091–0.987	**0.048**	0.370	0.108–1.266	0.113
CD3 after NAC (High/Low)	0.744	0.361–1.532	0.423			
CD4 after NAC (High/Low)	0.856	0.418–1.755	0.672			
CD8 after NAC (High/Low)	0.471	0.221–1.007	0.052	0.493	0.228–1.065	0.072

## Discussion

Clarifying the dynamic change in PD-L1 expression and TILs infiltration before and after NAC in cervical cancer is clinically relevant because it provides a rationale for use of immune checkpoint inhibitors in the adjuvant setting.

For cervical cancer, Immunohistochemistry staining is applied to test for the expression of PD-L1 on tumor specimens with different cutoff point. In Heeren’s study [11], PD-L1 positivity was observed in > 5% (used as cutoff) of the tumor cells in 54% of the squamous cell carcinomas. In Saglam’s study [9], PD-L1 expression (≥1%) was seen in 14 cases (64%) and diffuse expression (≥50%) in 5 cases (23%) among 22 poorly differentiated SCC samples. In Reddy’s study [12], 74 squamous cervical cancer patients were tested and 28(37.8%) cases had ≥50% PD-L1 expression whereas benign cervical tissues were negative. Similar with previous studies, in our series, 87.3% of cases had ≥1% PD-L1 expression, and 32.4% had ≥50% PD-L1 staining. The high expression cutoff point setting in our study is based on Garon’s NSCLC study [13]. In that clinical trial, patients with≥50% PD-L1 staining showed positive reactions to a targeted immune checkpoint inhibitor. The current one drug one predictive biomarker approach leads to each PD-1/PD-L1 inhibitor being associated with a unique PD-L1 antibody. To date, two mechanisms for the regulation of PD-L1 expression in tumor cells have been reported: innate immune regulation and adaptive immune regulation [14]. The former is the constitutive PD-L1 expression in tumor cells and the latter is an adaptive immune resistance to local inflammatory signals. In our patients before NAC, although PD-L1 expression was observed coexistence with lymphocytes in some areas, no significant correlations between tumor PD-L1 expression and CD3+, CD4+, CD8+ TILs was seen, which implied that the influence of local immune response to PD-L1 expression is limited at this time. Some basic researches showed that HPV infection likely plays a role in inducing PD-L1 expression. In their research, viral E6 or E7 genes could induce the PD-L1 expression [15, 16]. Similarly, a research in tonsillar cancer showed the PD-L1 positivity (> 1%) rate was 83.3% in HPV-positive cases and 56.9% in HPV-negative cases [17]. In our cases, 94.9% (111/117) patients were HPV positive and more HPV positive cases seemed to have high PD-L1 expression than HPV negative cases, although the difference was not significant. These researches along with our result suggested that innate immune regulation may play a more important role for PD-L1 expression in patients without chemotherapy.

To the best of our knowledge, this is the first study to assess the change of PD-L1 in paired samples from cervical cancers. We found PD-L1 tumor expression increased in a significant proportion of patients after NAC. Similar with us, in Meng’s research, cervical cancer patients with chemotherapy history had over expression of PD-L1 in tumor cells than patients without chemotherapy history [18]. The mechanism underlying the role of chemotherapy in regulation of PD-L1 expression isn’t clear. Interestingly, we found PD-L1 expression was associated with CD8+ T cells after NAC in our series. In adaptive immune resistance, PD-L1 expression in tumor cell is the response to IFN-γ production from CD8+ T cells. This result implies that for cervical cancer after NAC, PD-L1 expression was partly induced by adaptive immune resistance. In a research of breast cancer, researchers found paclitaxel were able to potentiate IFN-γ induced PD-L1 expression in breast cancer cells and increase PD-L1 mediated T cell apoptosis [19].

We estimated chemotherapy-induced cell death can release tumor associated antigens which would greatly increase the density of TILs. However, no significantly elevation of CD3+, CD4+, CD8+ TILs after NAC was seen in our series. This could be because although chemotherapy leads to new immunoreactions, the cytotoxic effect adversely impacts the immune cells. Cancers have been categorized into 4 different tumor microenvironments based on the presence of TILs and PD-L1 expression, among which, Type I tumors (PD-L1+, TILs+) are most likely to benefit from anti-PD-1/L1 blockade, as these tumors are warm tumors with pre-existing intratumor T cells [7]. The lymphocyte predominant microenvironment and the increased PD-L1 expression after chemotherapy in our series showed that patients after NAC are suitable for immunotherapy.

The prognostic value of PD-L1 expression has been investigated in various cancers without NAC history including cervical cancer. Some researchers suggested PD-L1 expression on cervical cancer was associated with poor survival [20]. While other reports indicated that PD-L1 expression in cervical neoplasms has no impact on survival [21]. Few studies have focused on the relationship between chemotherapy response, survival and PD-L1 expression. In epithelial ovarian cancer, esophageal squamous cell carcinoma, although increased PD-L1 expression was seen after NAC, no prognostic impact of PD-L1 expression was detected [22, 23]*.* In our study, patients with decreased PD-L1 expression after NACT seemed to have loner DFS, although the difference was not statistically significant in multivariate analysis. Similar results were seen from NSCLC, in Shin’s research, there was a tendency for patients with an increase in PD-L1 expression to have shorter survival [10]. The discordance may attribute to the tumor heterogeneity and the different chemotherapy drugs. Further studies are required to elucidate the relationship between PD-L1 expression and prognosis of cervical cancer patients with NAC.

Our study has several limitations. First, the usage of the non-in vitro diagnostic (IVD) clone was a limitation of this study. As a companion diagnostics antibody, clone 22C3 has been developed specifically for pembrolizumab in cervical cancer patients, while the antibody clone in our research was ZR3. For the difference of affinity, there may be discrepancy in staining intensity and positive proportion. Second, the pre-NAC samples were from cervical biopsy, while the post-NAC samples were obtained by surgical resection. The discrepancy in sample resource may result to systemic difference in PD-L1 count. Third, In clinical practice, combined positive score (CPS) of PD-L1 ≥ 1 or the presence of MHI is used for the patient selection with PD-1/PD-L1 inhibitors treatment. In our experiment, due to the poor inter-reader agreement for PD-L1 immune cells staining results, PD-L1 expression was assessed only on tumor cells. In lung cancer researchers also found there was highly concordant for PD-L1 tumor counting but not for stromal immune cell count [24].

## Conclusions

In conclusion, we demonstrated cisplatin based chemotherapy can increase PD-L1 expression in cervical cancer. After NAC, PD-L1 expression was correlated with high CD8+ TILs and a tendency to longer survival was seen in patients with decreased PD-L1 expression. The increased PD-L1 expression and a lymphocyte predominant microenvironment after chemotherapy provide new rationale for the combination anti PD-1/PD-L1 antibody in cervical cancer patients with NACT.

## Supplementary information

**Additional file 1.** The figures of TILs densities and survival curve according to TILs.

## Data Availability

The data supporting the conclusions are included in the article. Raw data are available upon request.

## Abbreviations

NAC: Neoadjuvant chemotherapy
SCC: Squamous cell carcinoma
TILs: Tumor infiltrating lymphocytes
PD-L1: Programmed death ligand 1
HPV: Human papillomatous virus
WHO: World health organization
DFS: Disease-free survival
