# Supplementary material for: Variation of PD-L1 expression in locally advanced cervical cancer following neoadjuvant chemotherapy
Source: Diagn Pathol. 2020 Jun 3;15:67. doi: 10.1186/s13000-020-00977-1 (PMC7271386; doi:10.1186/s13000-020-00977-1)
Supplement: Supplementary file 1 — Additional file 1. The figures of TILs densities and survival curve according to TILs. [file 13000_2020_977_MOESM1_ESM.pptx]

## Slide 1
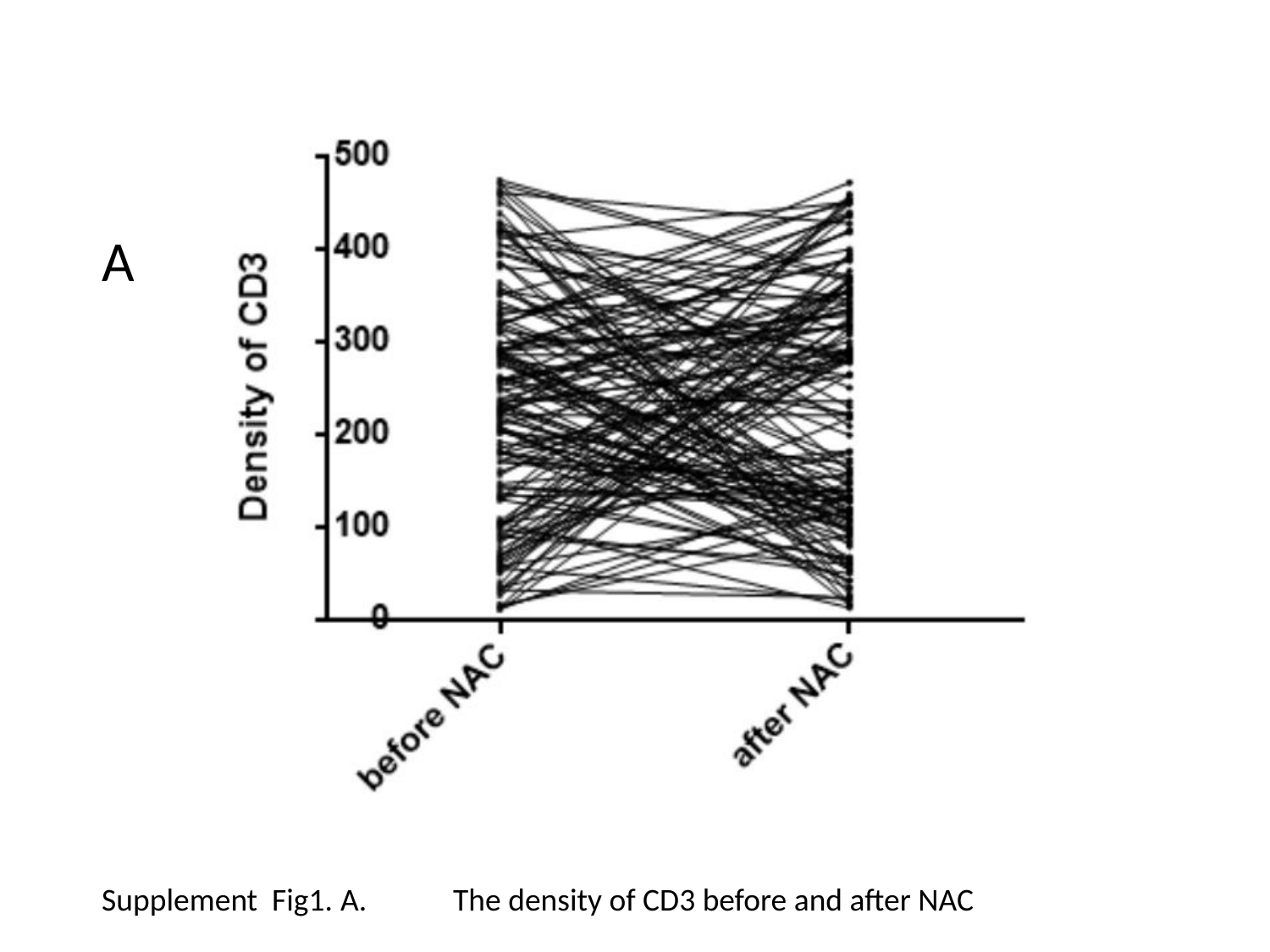

#
A
Supplement Fig1. A. The density of CD3 before and after NAC

## Slide 2
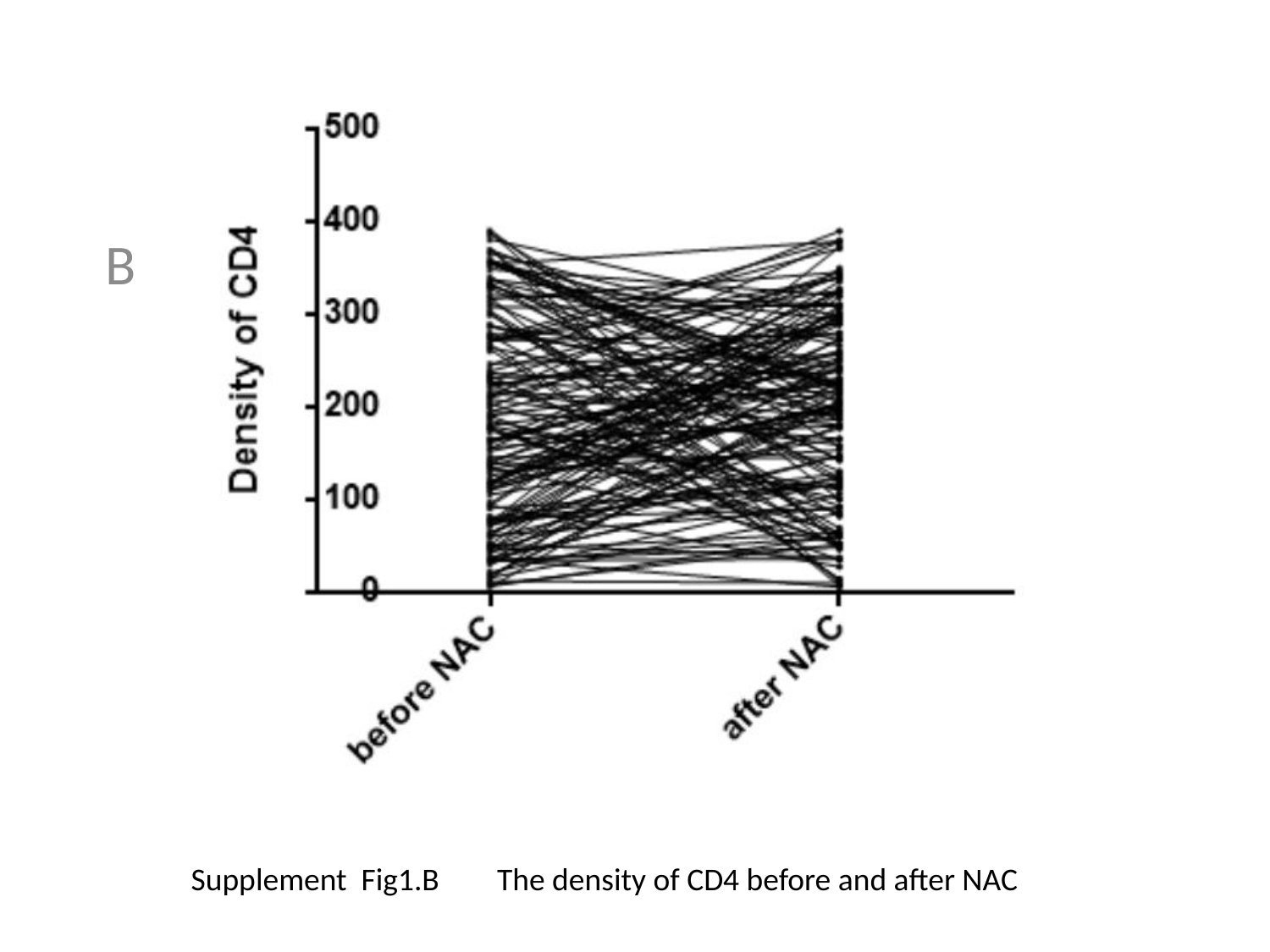

B
Supplement Fig1.B The density of CD4 before and after NAC

## Slide 3
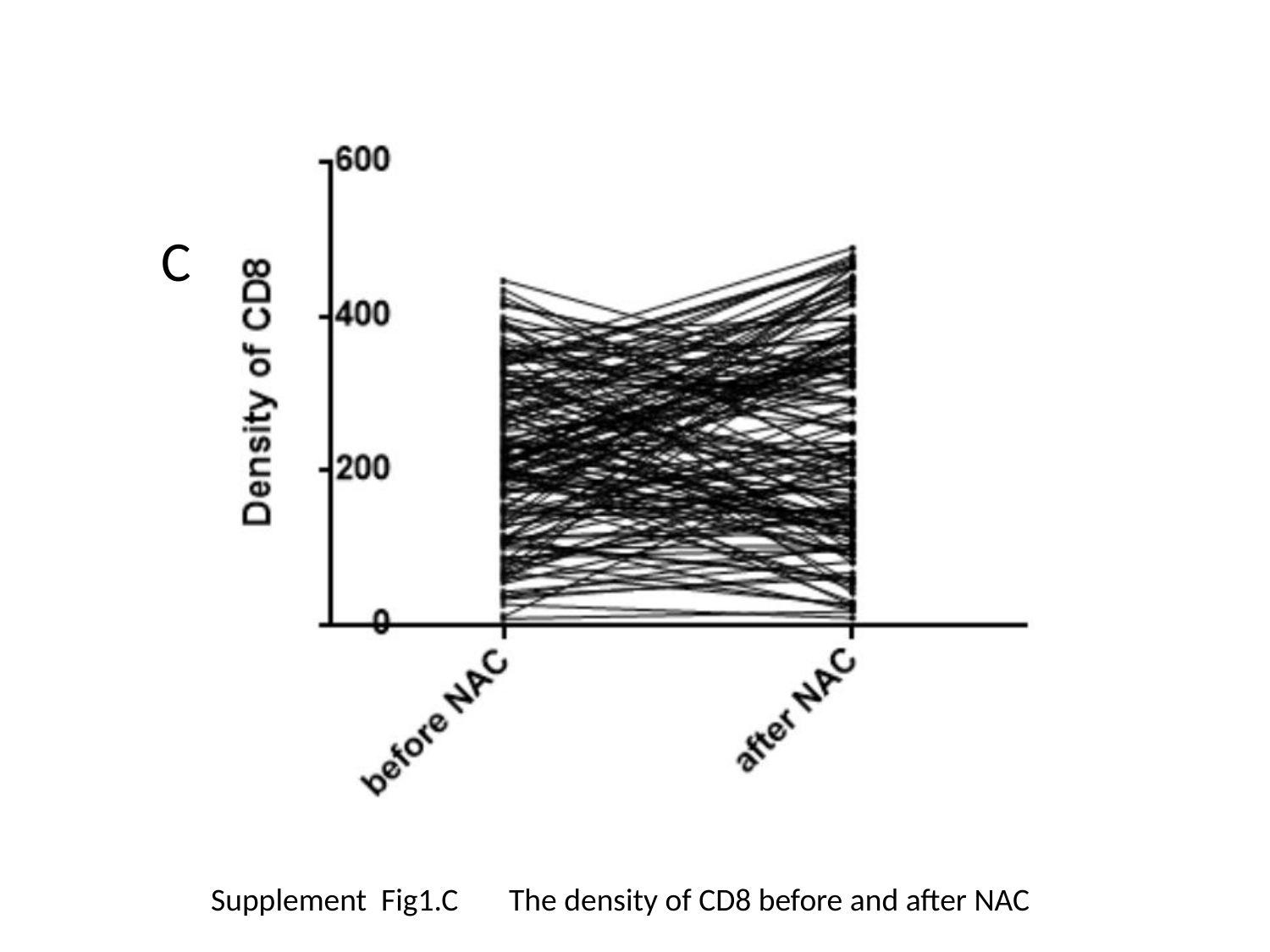

#
C
Supplement Fig1.C The density of CD8 before and after NAC

## Slide 4
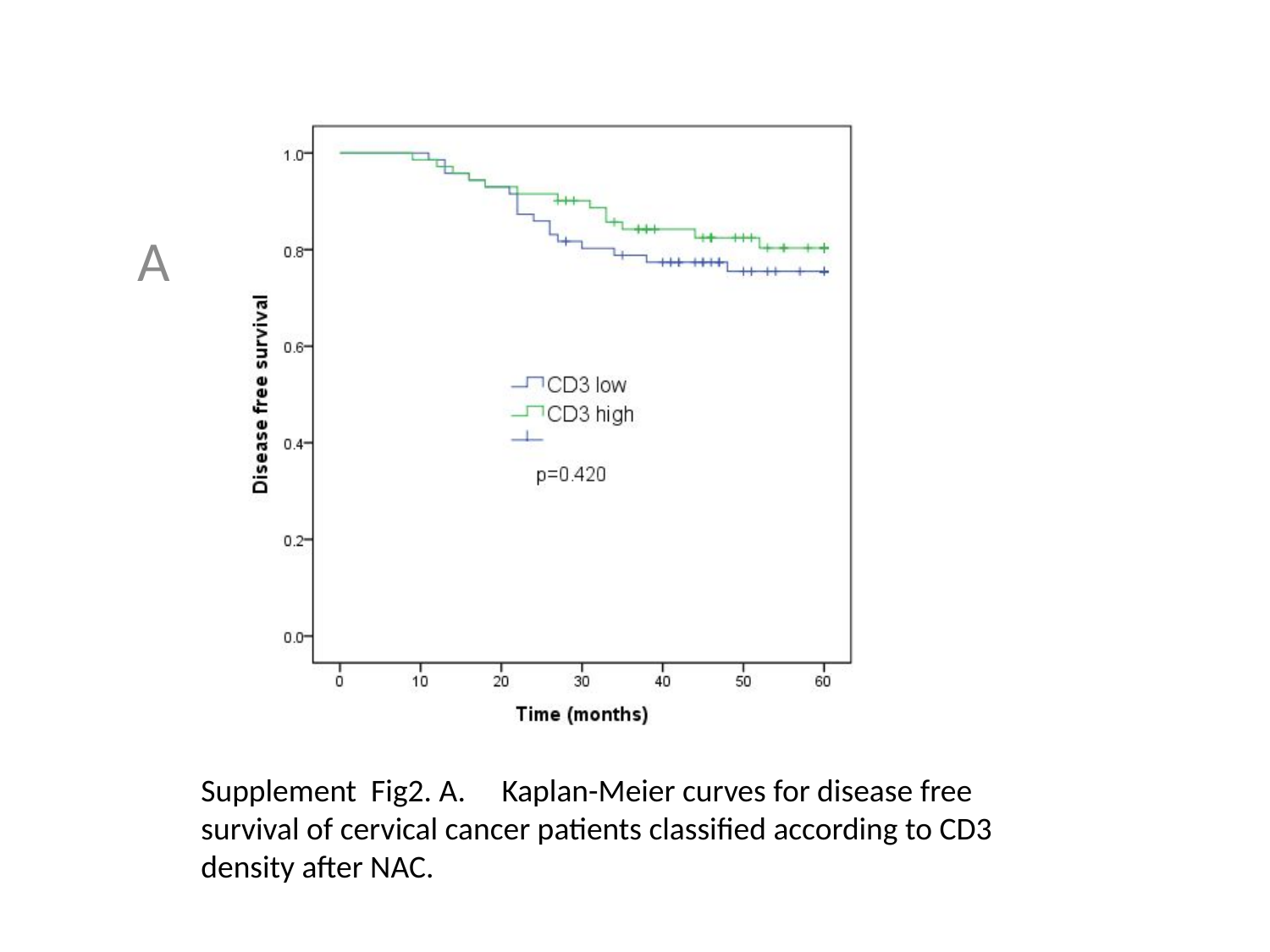

A
Supplement Fig2. A. Kaplan-Meier curves for disease free survival of cervical cancer patients classified according to CD3 density after NAC.

## Slide 5
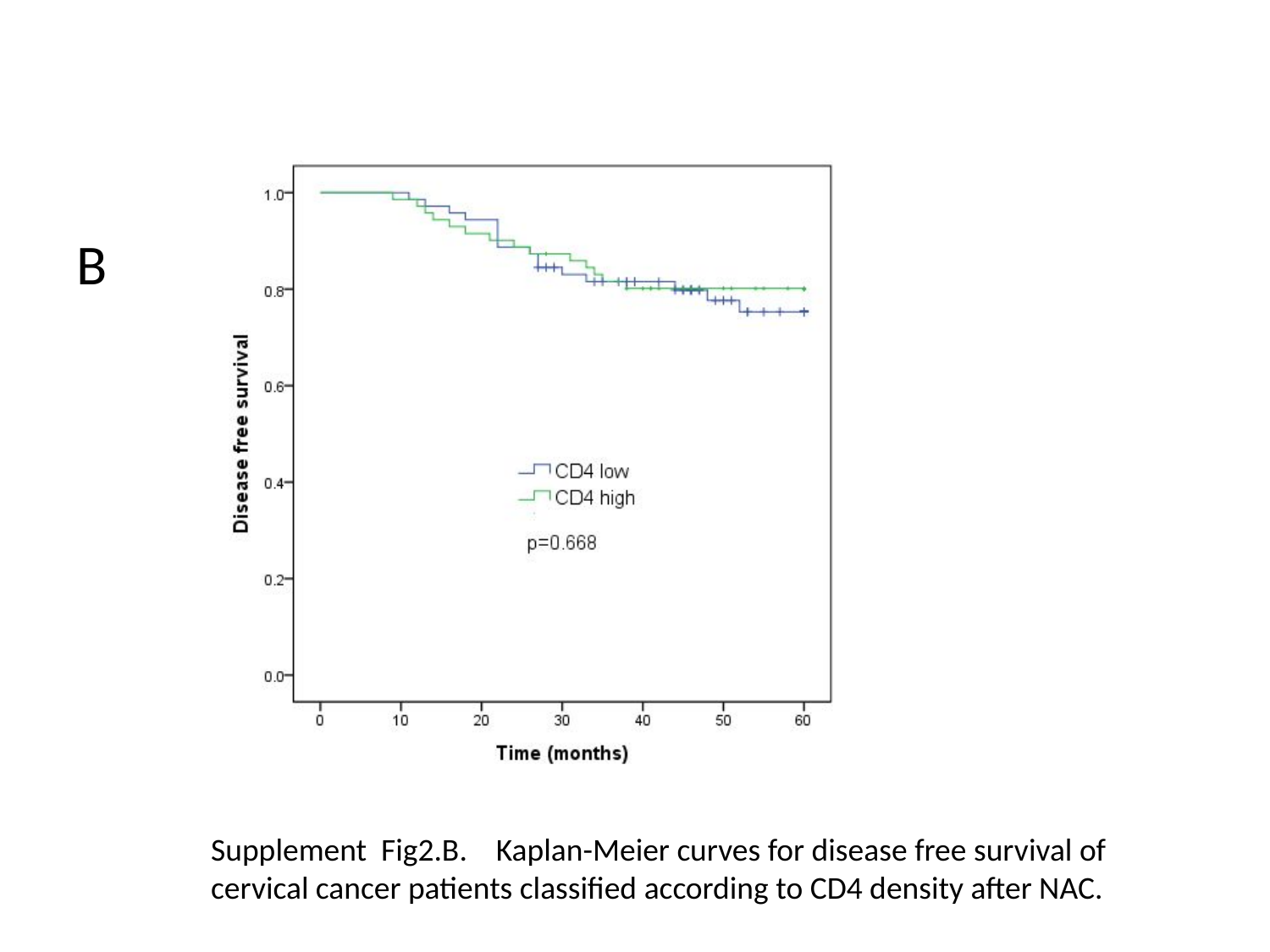

#
B
Supplement Fig2.B. Kaplan-Meier curves for disease free survival of cervical cancer patients classified according to CD4 density after NAC.

## Slide 6
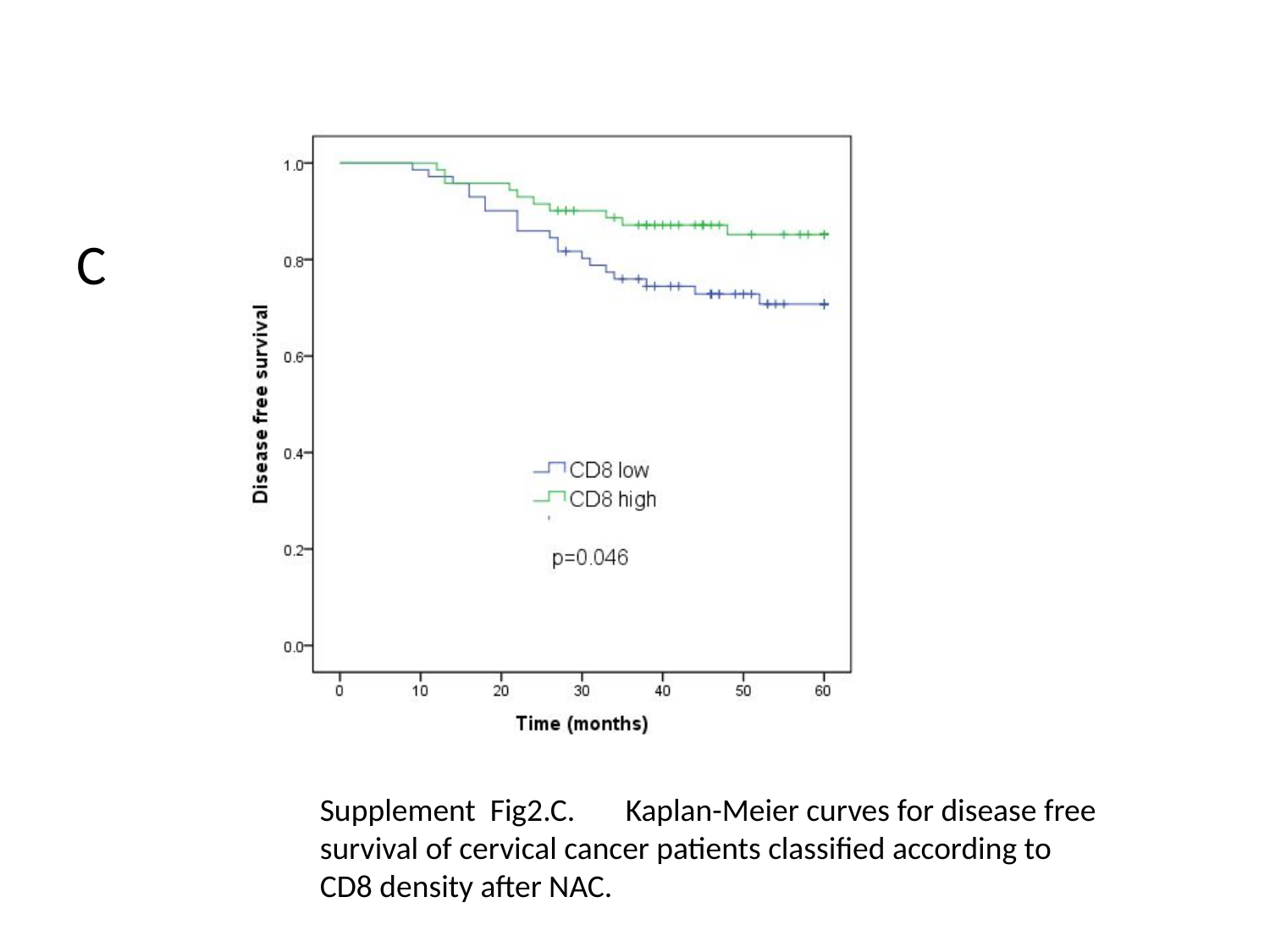

#
C
Supplement Fig2.C. Kaplan-Meier curves for disease free survival of cervical cancer patients classified according to CD8 density after NAC.
